# Treating Transthyretin Amyloidosis via Adeno-Associated Virus Vector Delivery of Meganucleases

**DOI:** 10.1089/hum.2022.061

**Published:** 2022-11-14

**Authors:** Jenny A. Greig, Camilo Breton, Scott N. Ashley, Kelly M. Martins, Cassandra Gorsuch, Joanna K. Chorazeczewski, Thomas Furmanak, Melanie K. Smith, Yanqing Zhu, Peter Bell, Wendy Shoop, Hui Li, Jeff Smith, Ginger Tomberlin, Peter Clark, Thomas W. Mitchell, Elizabeth L. Buza, Hanying Yan, Derek Jantz, James M. Wilson

**Affiliations:** ^1^ Gene Therapy Program, Perelman School of Medicine, University of Pennsylvania, Philadelphia, Pennsylvania, USA.; ^2^ Precision BioSciences, Inc., Durham, North Carolina, USA.

**Keywords:** transthyretin amyloidosis, transthyretin, meganuclease, AAV, gene therapy, gene editing

## Abstract

Transthyretin amyloidosis (ATTR) is a progressive and fatal disease caused by transthyretin (TTR) amyloid fibril accumulation in tissues, which disrupts organ function. As the TTR protein is primarily synthesized by the liver, liver transplantation can cure familial ATTR but is not an option for the predominant age-related wild-type ATTR. Approved treatment approaches include TTR stabilizers and an RNA-interference therapeutic, but these require regular re-administration. Gene editing could represent an effective one-time treatment. We evaluated adeno-associated virus (AAV) vector-delivered, gene-editing meganucleases to reduce TTR levels. We used engineered meganucleases targeting two different sites within the *TTR* gene. AAV vectors expressing TTR meganuclease transgenes were first tested in immunodeficient mice expressing the human *TTR* sequence delivered using an AAV vector and then against the endogenous *TTR* gene in rhesus macaques. Following a dose of 3 × 10^13^ genome copies per kilogram, we detected on-target editing efficiency of up to 45% insertions and deletions (indels) in the TTR genomic DNA locus and >80% indels in *TTR* RNA, with a concomitant decrease in serum TTR levels of >95% in macaques. The significant reduction in serum TTR levels following *TTR* gene editing indicates that this approach could be an effective treatment for ATTR.

## INTRODUCTION

Transthyretin amyloidosis (ATTR) is a rare, progressive fatal disease caused by accumulated transthyretin (TTR) amyloid fibrils in tissues. The tetrameric TTR protein is primarily synthesized in the liver. Dysfunctional TTR arising from mutation- or age-related issues with the wild-type protein becomes dissociated in the blood and generates misfolded monomers, which aggregate into amyloid fibrils that disrupt organ function.^1,2^ The process by which wild-type TTR becomes amyloidogenic with age is not well understood. However, the aggregation of wild-type TTR predominantly presents in men over 60 years of age as cardiomyopathy.^3^

The hereditable form of ATTR displays autosomal dominance with over 100 mutations known to result in amyloidosis.^4,5^ While there is considerable phenotypic heterogeneity in clinical manifestations, hereditary ATTR primarily manifests as polyneuropathy and cardiomyopathy. The most common mutation (V30M) is associated with polyneuropathy in ATTR patients. Other symptoms can include gastrointestinal disturbances and bilateral carpal tunnel syndrome.^5–7^ Genetic testing is necessary for determining the diagnosis and causative mutation. As TTR is primarily synthesized in the liver, liver transplantation can cure hereditary ATTR patients by ablating production of the circulating mutant TTR. However, organ transplantation requires immunosuppressive drugs to prevent rejection. Moreover, long-term outcomes following this procedure vary based on several factors such as the causative mutation.^8^

An alternative treatment approach involves TTR stabilizers, including tafamidis, to prevent dissociation of tetrameric TTR into its constituent monomers.^9^ While approved in the United States for the treatment of ATTR cardiomyopathy,^10^ tafamidis has variable efficacy in patients with later-stage polyneuropathy and may be mutation dependent for this symptom in hereditable ATTR.^11^

Recently, two TTR knockdown approaches have received approval for the treatment of ATTR polyneuropathy: inotersen and patisiran. These drugs have different mechanisms of action, but both knock down expression of mutant and wild-type TTR, thus making them effective for all ATTR manifestations by reducing circulating TTR by ∼80%.^12,13^ However, these drugs require regular re-administration. Inotersen is an antisense oligonucleotide therapy which binds to *TTR* mRNA and targets it for degradation^14^; it requires weekly subcutaneous injections. Moreover, this drug has safety concerns such as thrombocytopenia and glomerulonephritis.^13,15^ Patisiran is a small interfering RNA that also targets *TTR* mRNA for degradation and is administered intravenously every 3 weeks.^12^ Since this drug is delivered via lipid nanoparticle (LNP) to the liver, patients require premedication with corticosteroids, antihistamines, and acetaminophen to prevent infusion reactions. In contrast to these alternative treatments, a gene-editing approach could provide an effective one-time therapy.

A gene-editing approach utilizing liver-targeted LNPs for delivery of a CRISPR-Cas9-based strategy has shown reductions of >94% in TTR levels in mice and nonhuman primates (NHPs).^16,17^ Interim clinical trial data from this approach were recently published in which reductions of 87% (range of 80–96%, *n* = 3 subjects) were achieved in hereditary ATTR patients at day 28 following a dose of 0.3 mg/kg.^18^

We previously evaluated an adeno-associated virus (AAV) vector-delivered, liver-directed meganuclease for *in vivo* gene editing of the pro-protein convertase subtilisin/kexin type 9 (PCSK9) gene.^19–21^ PCSK9 is an antagonist of the low-density lipoprotein (LDL) receptor, meaning that a loss of function of PCSK9 reduces LDL levels; therefore, this approach could be useful in the treatment of hypercholesterolemia. The PCSK9 meganuclease recognized and generated a double-stranded break within a specific 22 bp sequence in the *PCSK9* gene, allowing endogenous DNA repair mechanisms to generate insertions and deletions (indels) at the cleavage site that disrupted PCSK9 protein expression. Following intravenous (IV) administration of various dose levels of AAV vectors in NHPs, circulating PCSK9 levels ranged from 16% to 83% of baseline levels for over 3 years.^20^

In this study, we evaluated an AAV-delivered, meganuclease-based, gene-editing approach to reduce TTR levels. We tested two different engineered meganucleases, each having a unique target site within the *TTR* gene. The most effective meganuclease produced a sustained >95% reduction from baseline of serum TTR levels in rhesus macaques. This significant reduction in serum TTR levels following genomic editing of the *TTR* gene indicates that AAV delivery of a TTR-specific meganuclease could represent an effective treatment for ATTR.

## MATERIALS AND METHODS

### Data availability statement

All data discussed in the article are available in the main text or Supplementary Materials. Complete clinical pathology data can be obtained upon request.

### AAV vector production

All AAV vectors were produced by the Penn Vector Core at the University of Pennsylvania as previously described.^22^ Briefly, plasmids expressing either the M1TTR or M2TTR engineered meganucleases from the thyroxine-binding globulin (TBG) promoter, including the WPRE sequence, were packaged within the AAV8 capsid. The M1TTR meganuclease targets a site in exon 1 within the TTR gene that is conserved in mouse, NHP, and human genomes, whereas the *TTR* exon 3 target site for the M2TTR meganuclease is not present in the mouse genome but is conserved in NHPs and humans.

### Mice

All animal procedures were performed in accordance with protocols approved by the Institutional Animal Care and Use Committee of the University of Pennsylvania. Male *Rag1*^−/−^ mice 6–8 weeks of age were purchased from The Jackson Laboratory (Bar Harbor, ME) and received an IV injection of 3 × 10^13^ genome copies per kilogram (GC/kg) of AAV8.TBG.hTTR(V30M) or vehicle control (phosphate-buffered saline [PBS]) on day 0 (*n* = 5/group). On day 14, mice were IV administered 3 × 10^11^ to 3 × 10^13^ GC/kg of AAV8.TBG.M1TTR or AAV8.TBG.M2TTR. Mice administered M1TTR were necropsied at day 56 (*n* = 5/group) and those administered M2TTR were necropsied on days 28 (*n* = 1), 42 (*n* = 2), and 56 (*n* = 2).

### Rhesus macaques

Wild-type rhesus macaques 3–6 years old (*n* = 8, one male [administered with the low dose of AAV8.TBG.M1TTR] and seven females) were obtained from Covance (Princeton, NJ). NHP studies were conducted at the University of Pennsylvania within a facility that is U.S. Department of Agriculture registered, Association for Assessment and Accreditation of Laboratory Animal Care accredited, and Public Health Service assured. As previously described,^23^ animals were housed in stainless steel cages with perches. All cage sizes and housing conditions were compliant with the Guide for the Care and Use of Laboratory Animals. A 12-h light/12-h dark cycle was maintained and controlled using an Edstrom Watchdog system.

Animals were fed Certified Primate Diet 5048 (PMI Feeds, Inc., Brentwood, MO) twice per day (morning and evening). An additional variety of food treats that were fit for human consumption, including fruits, vegetables, nuts, and cereals, were given daily as part of the standard enrichment process. Manipulanda such as kongs, mirrors, a puzzle feeder, and raisin balls were provided daily. Animals also received visual enrichment along with human interaction on a daily basis. All interventions were performed during the light cycle, and animals were fasted overnight before being anesthetized.

On study day 0, macaques were administered 10 mL of vector into the saphenous vein at a rate of 1 mL/min using an infusion pump (Harvard Apparatus, Holliston, MA). Two macaques each received a dose of 6 × 10^12^ and 3 × 10^13^ GC/kg of AAV8.TBG.M1TTR or AAV8.TBG.M2TTR (*n* = 2/dose/vector). From vector-administration day through weeks 8–12, all NHPs received prednisolone at a dose of 1 mg/kg/day orally, after which animals were tapered off prednisolone by gradual reduction of the daily dose.

### Analysis during the in-life phase

We anesthetized rhesus macaques and collected blood samples on selected days using the femoral vein. Antech (Irvine, CA) performed complete blood counts, clinical chemistries, and coagulation panels on the blood samples. Neutralizing antibody (NAb) titers were determined on serum samples taken before initiation of the study and throughout the in-life phase, as described previously.^24,25^ Macaques were also screened for the presence of the meganuclease target sites before vector administration on DNA extracted from peripheral blood mononuclear cells.

### Liver biopsy

On days 18 and 128 post-vector administration, a mini laparotomy procedure was performed to isolate liver tissue. Collected samples were divided into those for histopathology (*i.e.*, fixed in 10% neutral buffered formalin) or biodistribution (*i.e.*, frozen on dry ice and stored at −60°C or colder).

### Detection of TTR in serum

Blood was collected in serum separator tubes, allowed to clot, and serum was isolated. We analyzed mouse serum samples for both endogenous mouse levels and human TTR levels derived from the AAV8.TBG.hTTR(V30M) vector using enzyme-linked immunosorbent assay (ELISA) in accordance with the manufacturer's instructions. We determined mouse and human TTR levels using the Prealbumin ELISA Kit (mouse) from AVIVA Systems Biology (San Diego, CA) and PreAlbumin (TTR) ELISA Kit from Abcam (Cambridge, United Kingdom), respectively.

Charles River Laboratories quantitated the intact NHP TTR protein in serum using mass spectrometric immunoassay microcolumns (Thermo Fisher) and ultra-high-performance liquid chromatography–high-resolution mass spectrometry with a Q-Exactive Orbitrap mass spectrometer.

### Using next-generation sequencing for on- and off-target analysis

For on-target analysis of DNA extracted from mouse liver samples, we evaluated the indel % in the region of interest using Amplicon-Seq as previously described.^19–21^ Briefly, the region of interest within *TTR* exon 1 for M1TTR and exon 3 for M2TTR was amplified by polymerase chain reaction (PCR). We then generated next-generation sequencing (NGS) libraries from the PCR product, which were sequenced on a MiSeq instrument (Illumina, San Diego, CA). These sequences were then mapped to a reference genome (Assembly GRCm38.p6). Using a custom script, we quantified unedited reads and reads containing indels.^19–21^ The same analysis was performed for RNA, which was reverse transcribed to cDNA using random primers.

For NHP samples, we quantified AAV integration and translocations in addition to indels by AMP-Seq.^19–21,26^ Briefly, DNA was purified and sheared using a ME220 focused-ultrasonicator (Covaris, Woburn, MA) and purified with Agencourt AMPure XP beads (Beckman Coulter, Brea, CA). Fragments were end-repaired, A-tailed, and ligated to special adapters. We generated NGS libraries by two rounds of nested PCR using either the negative or positive primers. Libraries were sequenced on a MiSeq instrument, and the resulting sequences were mapped to a reference genome (Mmul_8.0.1 for rhesus macaque) and the administered AAV vector genome. We used a custom script to characterize and quantify edited alleles, as previously reported.^19–21^

To evaluate off-targets in NHP samples, we performed inverted terminal repeat (ITR)-Seq.^27^ Briefly, this unbiased genome-wide detection of off-targets was performed on purified liver DNA that was sheared using a ME220 focused ultrasonicator, end-repaired, A-tailed, and ligated to special adapters as described for AMP-Seq. Using AAV-ITR and adapter-specific primers, we amplified ITR-containing DNA fragments and generated NGS-compatible libraries. DNA was sequenced on a MiSeq instrument and the obtained reads were mapped to the rhesus macaque reference genome and the administered AAV vector genome. We used a custom script to identify off-target sites from the mapped reads, as previously described.^27^ A subset of genomic regions identified as off-target sites by ITR-Seq were sequenced by Amplicon-Seq; we then determined the indel %.

### Vector GC and transgene RNA analysis

Liver samples were snap-frozen at the time of biopsy or necropsy; we extracted DNA using the QIAamp DNA Mini Kit (Qiagen, Valencia, CA). DNase-treated total RNA was isolated from 100 mg of tissue. We quantified RNA by spectrophotometry, and aliquots were reverse transcribed to cDNA using random primers. We detected and quantified vector GC in extracted DNA and relative meganuclease transcript expression in extracted RNA using real-time PCR, as described previously.^23,28^ Briefly, vector GC and RNA levels were quantified using primers and probes designed against a vector-specific sequence, respectively.

### *In situ* hybridization and immunohistochemistry

Liver samples were fixed in 10% neutral buffered formalin and used to determine meganuclease expression by *in situ* hybridization (ISH) and immunohistochemistry (IHC). IHC was performed on sections obtained from formalin-fixed, paraffin-embedded liver samples. Sections were deparaffinized through a xylene and ethanol series followed by antigen retrieval using a pressure cooker (TintoRetriever Pressure Cooker; Bio SB) with 1 × Antigen Unmasking Solution (Vector Labs H-3300); boiling time was set to 20 min. We then treated the sections sequentially with 2% H_2_O_2_ (15 min; Sigma), avidin/biotin blocking reagents (15 min each; Vector Laboratories), and blocking buffer (1% donkey serum in PBS +0.2% Triton for 15 min) before incubating the sections with primary (45 min at 37°C) and biotinylated secondary antibodies (30 min at 37°C). The primary rabbit antibody against the meganuclease was provided by Precision BioSciences and used at a 1:2,000 dilution. Secondary antibody biotinylated donkey anti-rabbit antibodies were used at a 1:500 dilution (Jackson ImmunoResearch). All antibodies were diluted in 1% donkey serum.

After incubation, slides were washed in PBS, and a VECTASTAIN Elite ABC Kit (Vector Laboratories) was used according to the manufacturer's instructions with 3,3-diaminobenzidine as substrate to stain-bound antibodies. Sections were dehydrated through ethanol and xylene, and coverslips were applied.

Sections from formalin-fixed, paraffin-embedded liver tissues were used for ISH with the ViewRNA ISH Tissue Assay Kit (Life Technologies) according to the manufacturer's protocol. Z-shaped probe pairs binding to the meganuclease RNA were synthesized by the kit manufacturer. We visualized the location of hybridized probe sequences by the deposition of Fast Red substrate, which we imaged with a fluorescence microscope using a rhodamine filter set. Sections were counterstained with DAPI (4′,6-diamidino-2-phenylindole) to visualize nuclei.

We performed hematoxylin and eosin staining on sections from paraffin-embedded liver biopsy samples. Processing and staining procedures were conducted according to standard protocols. Liver sections were evaluated by a board-certified veterinary pathologist.

### Statistical analysis

We used linear mixed-effect modeling to evaluate significant differences in serum TTR levels over time. We compared mouse *TTR* RNA from individual groups using the Wilcoxon rank-sum test. On selected off-target sites, we statistically analyzed indel % using Fisher's Exact test to detect significant differences between peripheral blood mononuclear cells isolated before vector administration (Pre) and day 18 and Pre and day 128. All values are presented as mean ± standard error of the mean. A *p*-value of <0.05 was considered significant.

## RESULTS

### Reduction in serum TTR levels following gene editing in mice with a meganuclease that targets both murine and human *TTR*

We first evaluated the M1TTR meganuclease that targets a site that is conserved in mouse, NHP, and human genomes. To determine its editing potential on the mouse and human TTR sequences, we first administered an AAV8 vector expressing the V30M mutant version of the human *TTR* gene as a transgene. A similar strategy was previously employed to evaluate gene editing of the human *PCSK9* gene in mice.^19,21^

**Figure 1.**
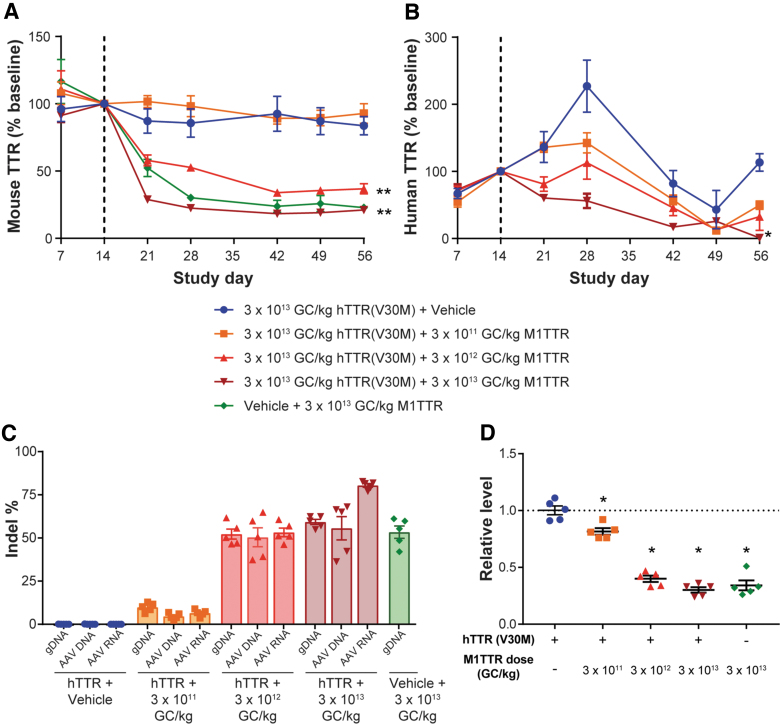
Dose-dependent editing and reduction in serum TTR levels following *in vivo* gene editing with a meganuclease that targets both mouse and human *TTR*. Male *Rag1^−/−^* mice were IV administered with 3 × 10^13^ GC/kg of AAV8.TBG.hTTR(V30M) or vehicle control on day 0 (*n* = 5/group). On day 14, mice were IV administered with 3 × 10^11^, 3 × 10^12^, and 3 × 10^13^ GC/kg of AAV8.TBG.M1TTR or vehicle control. Blood was collected at selected time points for serum mouse **(A)** and human TTR levels **(B)**, respectively. *Dashed line* indicates time of meganuclease vector administration (day 14). Percentage of baseline TTR levels was calculated using the average of day 14. Mice were necropsied on day 56. **(C)** Liver was harvested at necropsy for evaluation of indel % in mouse gDNA, the AAV genome (AAV DNA), and human *TTR* RNA derived from the first vector (AAV RNA). **(D)** Evaluation of relative expression of mouse *TTR* RNA transcript. Values presented as mean ± SEM. *p < 0.05, **p < 0.001. AAV, adeno-associated virus; GC, genome copy; gDNA, genomic DNA; hTTR, human transthyretin; indel; insertion and deletion; IV, intravenous; SEM, standard error of the mean; TTR, transthyretin.

Immunodeficient male *Rag1*^−/−^ mice were administered 3 × 10^13^ GC/kg of AAV8.TBG.hTTR(V30M) or vehicle control on day 0. Fourteen days later, mice were administered 3 × 10^11^, 3 × 10^12^, or 3 × 10^13^ GC/kg of AAV8.TBG.M1TTR or vehicle. Blood was collected throughout the in-life phase for evaluation of both mouse (Fig. 1A) and human (Fig. 1B) serum TTR protein levels, presented as percent of baseline TTR levels calculated from the average of the day 14 values. Mice administered vehicle or the lowest dose of vector expressing the meganuclease (3 × 10^11^ GC/kg) did not show any change in baseline mouse TTR levels (Fig. 1A). The mid and high doses of meganuclease vector (3 × 10^12^ and 3 × 10^13^ GC/kg) showed a significant reduction in mouse TTR levels of 63% and 79%, respectively; this effect was not influenced by preadministration with the AAV8.TBG.hTTR(V30M) expression vector or vehicle (Fig. 1A, *p* < 0.001).

Preadministration of the AAV8.TBG.hTTR(V30M) expression vector enabled evaluation of the editing potential of the M1TTR meganuclease for the human *TTR* gene. In mice administered 3 × 10^13^ GC/kg of AAV8.TBG.hTTR(V30M) on day 0 and then vehicle on day 14, expression of human TTR continued to increase to peak expression at day 28 and then plateaued at 113% of baseline by day 56 (Fig. 1B). Following administration of 3 × 10^11^, 3 × 10^12^, and 3 × 10^13^ GC/kg of the meganuclease vector, there was a dose-dependent decrease in human TTR levels to 50%, 33%, and <1% of baseline by day 56, respectively (Fig. 1B).

### Dose-dependent editing in both mouse genomic and vector DNA and RNA following *in vivo* gene editing

At day 56, mice were necropsied and livers harvested for evaluation of indels in (i) mouse genomic DNA (denoted as gDNA in Fig. 1C); (ii) the AAV vector genome (AAV DNA); and (iii) the human *TTR* RNA derived from the AAV8.TBG.hTTR(V30M) expression vector (AAV RNA, Fig. 1C). We observed only 5–10% indels in gDNA, AAV DNA, and AAV RNA in mice that received the lowest dose of meganuclease vector (3 × 10^11^ GC/kg). We saw indels of 50–53% across the mouse genomic DNA, AAV DNA, and AAV RNA for mice that received 3 × 10^12^ GC/kg. By contrast, mice administered 3 × 10^13^ GC/kg had 56–59% indels in the DNA analyses, with 80% indels observed in the vector-derived RNA (Fig. 1C). Indels were nearly identical (∼56%) in the mouse genome of animals administered either the AAV8.TBG.hTTR(V30M) expression vector or vehicle before 3 × 10^13^ GC/kg of the meganuclease vector. When we characterized the indels seen with the M1TTR meganuclease in both the gDNA and AAV DNA, we observed that the most common indel was a 1 bp deletion (data not shown).

We also evaluated changes in the relative expression of mouse *TTR* RNA where a dose-dependent decrease from an 18% reduction at the lowest meganuclease vector dose to a 70% reduction following administration of 3 × 10^13^ GC/kg was detected (Fig. 1D). We attributed the reduction in RNA levels to transcription of indels in the DNA sequence resulting in reduced expression and/or increased RNA degradation.

### Enhanced editing efficiency with a meganuclease targeting an alternative site within the TTR gene

**Figure 2.**
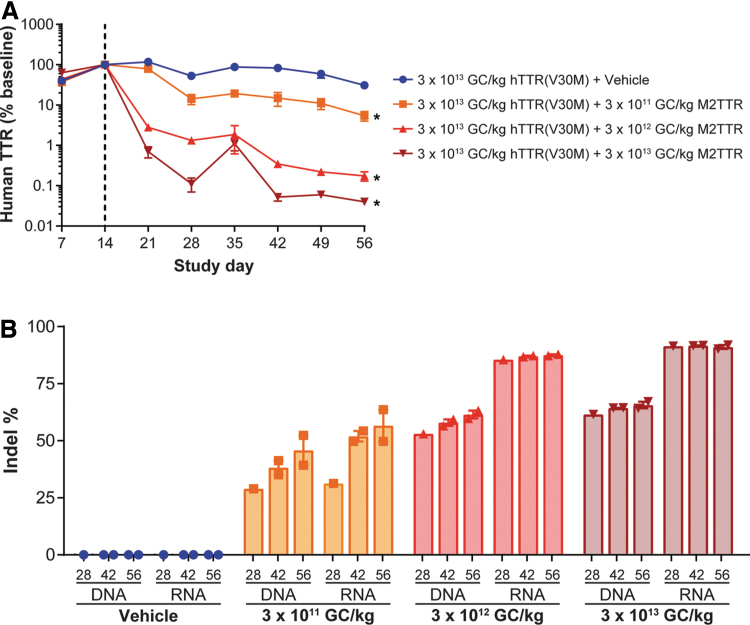
Gene editing of human *TTR* in mice results in reduced serum human TTR levels. Male *Rag1^−^*^/*−*^ mice were IV administered 3 × 10^13^ GC/kg AAV8.TBG.hTTR(V30M) on day 0 (*n* = 5 per group). On day 14, mice were IV administered 3 × 10^11^, 3 × 10^12^, or 3 × 10^13^ GC/kg AAV8.TBG.M2TTR. Blood was collected at selected time points for human TTR levels **(A)**. *Dashed line* indicates time of meganuclease vector administration (day 14). We calculated the percentage of baseline TTR levels using the average of day 14. Mice were necropsied on days 28 (*n* = 1), 42 (*n* = 2), and 56 (*n* = 2). **(B)** Livers were harvested at necropsy for evaluation of indel % in the AAV genome (DNA) and human TTR RNA derived from the first vector (RNA). Values presented as mean ± SEM. *p < 0.001.

An alternative engineered meganuclease—M2TTR—was designed to target a different sequence within the TTR gene compared to that of the M1TTR meganuclease. The target site for the M2TTR meganuclease is not present in the mouse genome but is conserved between NHPs and humans. To evaluate the *in vivo* efficacy of this meganuclease, we preadministered immunodeficient male *Rag1*^−/−^ mice with 3 × 10^13^ GC/kg of AAV8.TBG.hTTR(V30M) to supply the human *TTR* sequence on day 0. Blood was collected throughout the in-life phase for evaluation of human serum TTR levels (Fig. 2A). We presented these levels as percent of baseline TTR levels calculated from the average of the day 14 values. Expression of human TTR in mice administered vehicle rather than a vector expressing the meganuclease rose to a peak of 117% of baseline at day 21 post-vector administration. Expression then decreased to 31% of baseline by day 56 (Fig. 2A). There was a significant dose-dependent decrease in human TTR levels of 94.5%, 99.8%, and 99.9% following IV administration of 3 × 10^11^, 3 × 10^12^, and 3 × 10^13^ GC/kg of AAV8.TBG.M2TTR, respectively (Fig. 2A, *p* < 0.001).

At day 56, mice were necropsied and livers harvested for evaluation of indel % in the AAV vector genome and in the human *TTR* RNA derived from the AAV8.TBG.hTTR(V30M) expression vector (Fig. 2B). Again, there was a clear dose-dependent increase in gene editing within the AAV-supplied human *TTR* sequence at both the DNA and RNA level with up to 66% and 91% editing, respectively, at day 56 following a dose of 3 × 10^13^ GC/kg of AAV8.TBG.M2TTR. While the indel percentage increased over time for the low- and mid-dose groups, mice administered the highest dose of the meganuclease-expressing vector (3 × 10^13^ GC/kg) did not change from day 28 through day 56, suggesting maximal editing efficiency was achieved early in the study (Fig. 2B).

### Translation of gene editing potential from mice to NHPs

**Figure 3.**
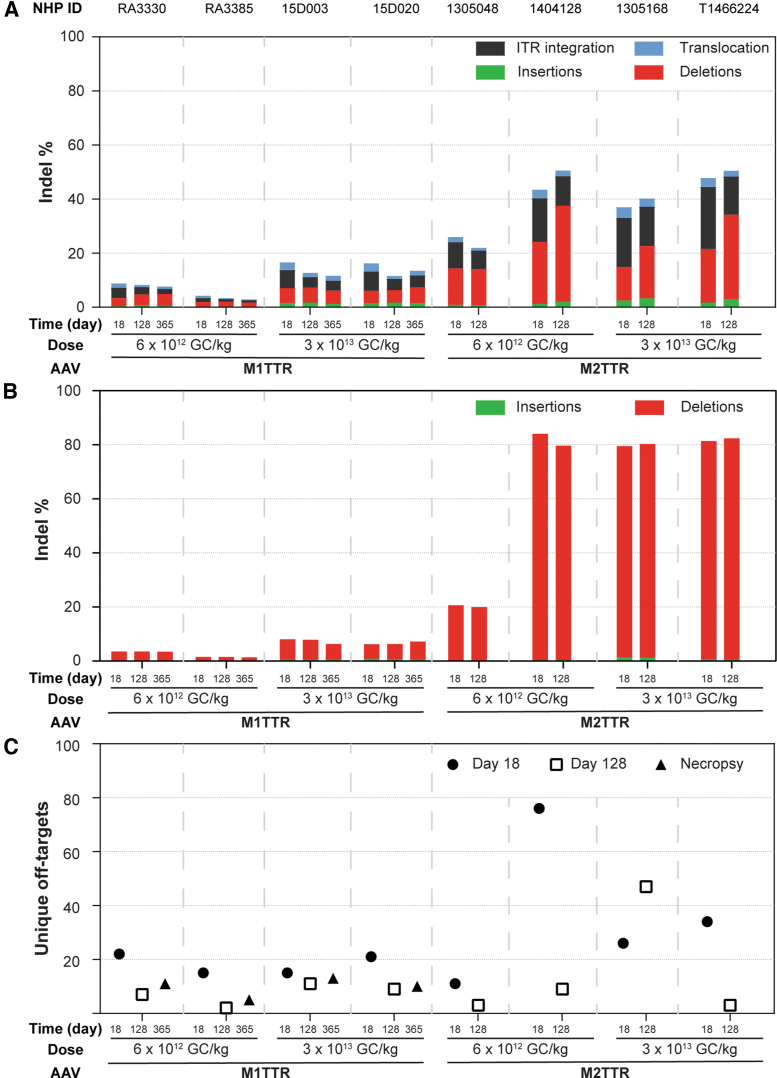
Translation of TTR gene editing from mice to NHPs. Rhesus macaques were IV administered 6 × 10^12^ and 3 × 10^13^ GC/kg of AAV8.TBG.M1TTR or AAV8.TBG.M2TTR. We performed liver biopsies on day 18 and 128 postvector administration. Animals administered with AAV8.TBG.M1TTR were necropsied 1-year postvector administration. We extracted DNA and RNA and performed NGS analysis, including AMP-Seq on DNA **(A)**, Amplicon-Seq on RNA **(B)**, and ITR-Seq on DNA **(C)**. ITR, inverted terminal repeat; NGS, next-generation sequencing; NHP, nonhuman primate.

The same engineered meganucleases (M1TTR and M2TTR) were then evaluated in rhesus macaques to determine the translatability of gene-editing efficacy. Rhesus macaques were IV administered 6 × 10^12^ or 3 × 10^13^ GC/kg of AAV8 vector expressing either the M1TTR or M2TTR meganuclease. All NHPs received prednisolone at a dose of 1 mg/kg/day orally for 8–12 weeks post-vector administration, at which point animals were tapered off prednisolone by a gradual reduction of daily dose. Liver biopsies were performed on day 18 and approximately day 128 post-vector administration to evaluate the indel frequency in the *TTR* locus of NHP genomic DNA and RNA by AMP-Seq and Amplicon-Seq, respectively, as well as off-targets by ITR-Seq (Fig. 3).

During the in-life phase of the study, NHPs were routinely monitored by blood draws for complete blood cell counts and serum chemistries. Some of the NHPs receiving either vector exhibited peaks in aspartate transaminase or alanine aminotransferase levels that mostly coincided with the time of the liver biopsy procedure (see Supplementary Fig. S1 for NHPs administered AAV8.TBG.M1TTR and Supplementary Fig. S2 for NHPs administered AAV8.TBG.M2TTR). Anti-AAV8 NAbs were also measured throughout the in-life phase for NHPs administered AAV8.TBG.M2TTR (Supplementary Fig. S3).

Assessment of editing in genomic DNA extracted from liver samples was performed by AMP-Seq, which evaluated insertions, deletions, ITR-integration (integration of vector sequences), and translocations (Fig. 3A). Macaques administered the M1TTR meganuclease had an average of 7% and 16% editing in genomic DNA on day 18 following administration of 6 × 10^12^ or 3 × 10^13^ GC/kg vector, respectively (Fig. 3A). Similarly, we observed a limited degree of editing in the *TTR* RNA of 2.5% and 7% at the low and high vector doses (Fig. 3B). Similar to our observations in mice, the indels generated by the M1TTR meganuclease in genomic DNA predominantly comprised a 1 bp deletion (Supplementary Fig. S4). Following this low level of on-target editing, the off-targets detected for this meganuclease constituted fewer than 22 unique sites across the time points evaluated (Fig. 3C).

In comparison, macaques administered vector expressing the M2TTR meganuclease showed high efficacy with little-to-no dose effect (Fig. 3). This observation excludes the reduced efficacy seen in one macaque administered the low dose of 6 × 10^12^ GC/kg of AAV8.TBG.M2TTR (26% editing in genomic DNA and 21% in RNA, Fig. 3A, B). NHPs administered AAV8.TBG.M2TTR showed 43% and 42% editing at day 18 in the genomic DNA and 84% and 80% editing in RNA at the low- and high-vector doses, respectively (Fig. 3A, B). This editing efficacy persisted between the two liver biopsy time points (day 18 and 128).

While the editing efficiency was slightly reduced from mice to NHPs at the RNA level following administration of 3 × 10^13^ GC/kg AAV8.TBG.M2TTR (91% in mice [Fig. 2B] vs. 80% in macaques [Fig. 3B]), these data illustrate more efficient translation between species for this vector-administered gene-editing approach than is usually seen for gene therapy-based applications. After characterizing the M2TTR indels, we found that this meganuclease resulted in similar levels of 2 and 3 bp deletions (Supplementary Fig. S5); a 3 bp deletion predominated in the *TTR* RNA (Supplementary Fig. S6).

The M2TTR meganuclease generated relatively few off-target events, as detected through ITR-Seq, in conjunction with its high-efficiency on-target editing activity (Fig. 3C). There was an average of 37 unique off-target sites across all NHPs injected with this meganuclease, with a maximum of 76 sites being detected in a single NHP. These off-target sites occurred more frequently within genes (>65%) than in intergenic regions, with a bias toward genes that are highly expressed in the liver (Supplementary Fig. S7).

We further validated the M2TTR off-target sites in the liver, in comparison to DNA extracted from peripheral blood mononuclear cells harvested before vector administration (Table 1). We amplified and sequenced the 19 top-ranking off-target sites identified by the ITR-Seq analysis to evaluate differences between day 18 and/or 128 and before vector administration (Pre). Statistically significant differences between Pre samples and either day 18 or 128 or between day 18 and 128 were evaluated by Fisher's Exact test (*p* < 0.05, Table 1). The number of off-target sites reduced from day 18 to 128 on average (from 37 to 16 unique sites), similar to that observed for the M2PCSK9 meganuclease.^19–21^ One of the off-target sites (chr8:11196161–11196201) had an average of 2.4% indels (range of 0.37–3.72% on day 18 post-vector administration, Table 1). The most likely off-target sequence in this region shares 19/22 bp homology to the target site for the M2TTR meganuclease, but the site is not conserved in the human genome.

**Table 1.** Off-target validation in liver after systemic administration of AAV8.TBG.M2TTR

	6 × 10^12^ GC/kg						3 × 10^13^ GC/kg					
	1305048			1404128			1305168			T1466224		
	Pre	d18	d128	Pre	d18	d128	Pre	d18	d128	Pre	d18	d128
chr12:87904928-87904968	0.09	**0.32**	0.09^*^	0.10	**1.21**	0.51^*^	0.09	**1.03**	0.83^*^	0.10	**0.27**	0.36^*^
chr8:11196161-11196201	0.21	**0.37**	0.18^*^	0.41	**3.72**	2.41^*^	0.23	**3.22**	3.78^*^	0.13	**2.35**	1.42^*^
chr5:37071902-37071942	0.06	**0.10**	0.05^*^	0.05	**0.27**	0.19^*^	0.05	**0.45**	0.36^*^	0.05	**0.29**	0.18^*^
chr16:45654359-45654399	0.04	**0.06**	0.03^*^	0.03	**0.11**	0.15^*^	0.03	**0.08**	**0.10**	0.03	**0.16**	0.06^*^
chr1:131368022-131368062	0.05	0.06	0.06	0.06	**0.11**	**0.11**	0.06	**0.12**	**0.10**	0.05	**0.07**	0.08^*^
chr5:65826942-65826982	0.05	0.08	0.04	0.05	**0.17**	0.04^*^	0.00	**0.20**	**0.20**	0.04	**0.15**	0.08^*^
chr2:95972521-95972562	0.04	0.04	0.04	0.05	**0.08**	0.05^*^	0.05	**0.11**	0.04^*^	0.05	0.05	0.05
chr6:1350225-1350265	0.05	0.05	0.08^*^	0.05	**0.24**	0.07^*^	0.05	**0.30**	0.41^*^	0.05	**0.17**	0.05^*^
chr5:54623831-54623871	0.06	0.09	0.04^*^	0.05	**0.20**	**0.17**	0.03	**0.25**	0.19^*^	0.06	**0.18**	0.07^*^
chr2:40480165-40480205	0.05	0.05	0.05	0.05	**0.10**	0.07^*^	0.06	**0.12**	**0.11**	0.05	0.05	**0.07**
chr16:75546540-75546581	0.04	0.03	0.04	0.04	**0.09**	0.03^*^	0.03	0.03	0.03	0.04	0.04	0.05
chr4:38395097-38395136	0.03	0.04	0.03	0.03	0.03	0.03	0.03	**0.08**	0.05^*^	0.03	**0.04**	0.03
chr17:74722587-74722627	0.07	0.07	0.07	0.07	**0.38**	0.21^*^	0.09	**0.33**	0.25^*^	0.07	**0.15**	**0.14**
chr16:26937570-26937610	0.05	**0.08**	**0.08**	0.07	0.06	0.07	0.07	0.07	0.08	0.06	**0.10**	0.07^*^
chr15:27727216-27727256	0.05	0.05	0.06	0.05	0.05	0.04	0.05	0.05	0.04	0.06	0.06	0.05
chr7:56260476-56260515	0.07	0.06	0.07	0.06	**0.11**	**0.09**	0.06	**0.10**	0.07^*^	0.08	0.08	0.11^*^
chr11:122161601-122161640	0.07	**0.10**	0.08^*^	0.07	0.08	0.08	0.08	**0.13**	**0.12**	0.10	**0.16**	0.12^*^
chr1:54693640-54693689	0.13	0.10	0.06	0.08	0.15	0.16	0.09	0.10	0.09	0.11	0.12	0.14
chr13:55676638-55676677	0.08	0.07	0.05	0.09	0.11	0.07^*^	0.08	0.12	0.13	0.07	**0.14**	0.09^*^

Rhesus macaques were IV administered 6 × 10^12^ or 3 × 10^13^ GC/kg of AAV8.TBG.M2TTR. We performed liver biopsies on day 18 (d18) and day 128 (d128) postvector administration. We extracted DNA from liver biopsy samples and PMBCs isolated before vector administration (Pre). We sequenced a subset of genomic regions identified as off-target sites by ITR-Seq and evaluated the indel %. Fisher's Exact test (*p* < 0.05) was used to perform statistical analysis. Bold typeface indicates significant differences between Pre and d18 and Pre and d128.

^*^ Indicates significant differences between day 18 and day 128.

GC, genome copies; indel; insertion and deletion; IV, intravenous; ITR, inverted terminal repeat.

**Figure 4.**
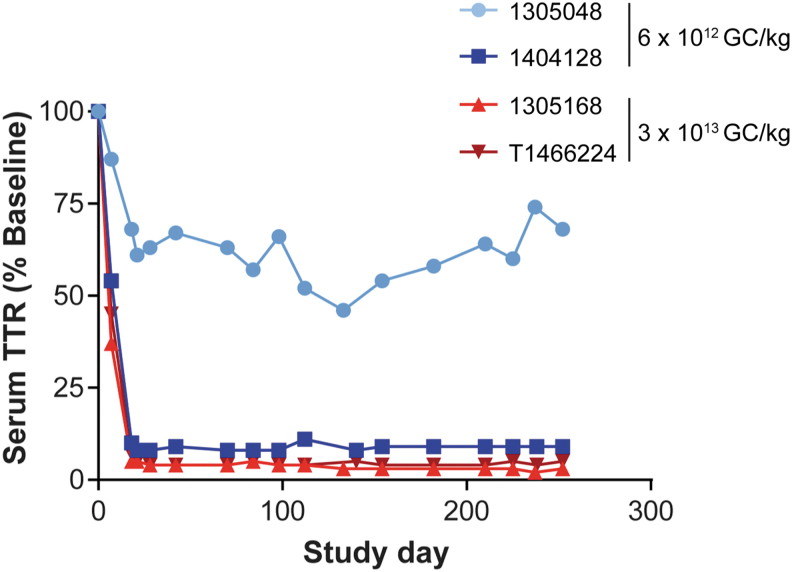
Substantial reduction in serum TTR levels in NHPs following systemic administration of AAV8.TBG.M2TTR. Rhesus macaques were IV administered 6 × 10^12^ or 3 × 10^13^ GC/kg of AAV8.TBG.M2TTR. We collected blood samples at selected time points for TTR levels, which are presented as percent of baseline levels.

We also evaluated the serum TTR levels in NHPs injected with the M2TTR meganuclease-expressing vector (Fig. 4). Both macaques administered the high-vector dose (3 × 10^13^ GC/kg) had a >95% reduction in serum TTR levels. One of the NHPs administered 6 × 10^12^ GC/kg had a decrease in serum TTR levels to 9% of baseline, whereas the other macaque dosed at 6 × 10^12^ GC/kg had an average plateau level of 61% of baseline (Fig. 4). When vector GC and meganuclease RNA transcript levels were evaluated on liver samples taken at biopsy, we found that the macaque with the partial reduction in serum TTR levels exhibited the lowest vector GC and RNA levels in the liver (Table 2), suggesting reduced gene-transfer efficiency.

**Table 2.** Liver vector genome copies and meganuclease RNA levels in liver following systemic administration of AAV8.TBG.M1TTR or AAV8.TBG.M2TTR

Vector	NHP ID	Dose, GC/kg	Study day	Vector GC/μg DNA	Vector GC/diploid genome	Transcript RNA/100 ng
AAV8.TBG.M1TTR	RA3330	6.00E+12	18	2.64E+06	13.20	3.37E+06
			128	1.18E+06	5.89	1.44E+04
			364	8.31E+04	0.42	6.60E+02
AAV8.TBG.M1TTR	RA3385	6.00E+12	18	1.83E+06	9.14	6.72E+05
			128	1.45E+06	7.27	5.55E+03
			364	1.19E+05	0.59	2.53E+02
AAV8.TBG.M1TTR	15D003	3.00E+13	18	1.72E+07	85.94	3.59E+06
			128	3.69E+06	18.44	8.55E+04
			364	3.51E+05	1.75	2.02E+03
AAV8.TBG.M1TTR	15D020	3.00E+13	18	1.23E+07	61.62	2.62E+06
			128	2.78E+06	13.90	4.91E+04
			364	1.18E+05	0.59	1.95E+03
AAV8.TBG.M2TTR	1305048	6.00E+12	18	1.77E+06	8.84	1.68E+05
			130	2.46E+05	1.23	3.68E+03
AAV8.TBG.M2TTR	1404128	6.00E+12	18	1.92E+06	9.59	4.35E+05
			137	1.49E+05	0.75	2.64E+04
AAV8.TBG.M2TTR	1305168	3.00E+13	18	8.00E+06	40.02	3.66E+05
			130	1.60E+06	8.01	9.04E+04
AAV8.TBG.M2TTR	T1466224	3.00E+13	18	1.76E+07	88.13	5.05E+05
			140	1.30E+06	6.51	3.90E+04

Rhesus macaques were IV administered 6 × 10^12^ or 3 × 10^13^ GC/kg AAV8.TBG.M1TTR or AAV8.TBG.M2TTR. We performed liver biopsies on day 18 and 128 postvector administration. Animals administered AAV8.TBG.M1TTR were necropsied 1-year postvector administration. We extracted DNA and RNA and determined vector GCs and meganuclease RNA levels.

NHP, nonhuman primate.

We also evaluated the liver biopsy samples from macaques administered AAV8.TBG.M2TTR for meganuclease expression at both the RNA and protein levels by ISH and IHC, respectively (Supplementary Fig. S8). We detected M2TTR meganuclease RNA and protein expression following either vector dose at day 18 postvector administration. Protein expression was undetectable by day 128 via IHC, however, consistent with previous observations of other meganuclease-expressing vectors.^19–21^ We also evaluated liver biopsy sections for histopathology by a board-certified veterinary pathologist (Supplementary Fig. S9). While there were reports of mononuclear cell infiltrates, these were minimal. The only other finding was a mild cytoplasmic hepatocellular vacuolation noted in one animal administered the high vector dose.

## DISCUSSION

We evaluated an AAV-delivered, meganuclease-based, gene-editing approach to reduce TTR levels as a potential treatment for ATTR. In this study, the selection of engineered meganucleases as the gene-editing platform enabled the administration of a single AAV to achieve editing of the *TTR* gene. This was possible because the M1TTR and M2TTR meganuclease coding sequences are small and easily packaged within a single AAV genome, with the M1TTR and M2TTR meganucleases being expressed from a single transcript. Our initial experiments were performed in immunodeficient mice to evaluate meganucleases that targeted sites within the mouse and/or human genome by providing the human *TTR* sequence expressed from an AAV vector and subsequent administration with meganuclease-expressing AAV vectors.

The M1TTR meganuclease demonstrated editing in both the endogenous mouse genomic *TTR* sequence and the exogenous human *TTR* transgene sequence supplied via AAV vector to similar levels across the dose range evaluated. The vector containing the human *TTR* sequence was dosed at 3 × 10^13^ GC/kg, which resulted in almost all hepatocytes being transduced with an average of 9 vector GC per cell. This high transduction likely resulted in the similar level of editing of both the endogenous mouse genomic *TTR* sequence and the exogenous human *TTR* sequence as they were both present in the same number of cells. While the lowest vector dose (3 × 10^11^ GC/kg) resulted in 10% indels in mouse genomic DNA (Fig. 1C) and an 18% reduction in mouse *TTR* RNA levels (Fig. 1D), we did not detect any change in mouse TTR levels (Fig. 1A). The same dose led to AAV-derived DNA and RNA editing by 4.6% and 6.5%, respectively (Fig. 1C), which, in turn, reduced vector-derived human TTR levels by 50%. This suggests possible differences in editing potential for genomic (mouse) and (likely) episomal (*i.e.*, human *TTR* derived from an AAV vector) DNA, endogenous mouse RNA stability and/or degradation compared to vector-derived RNA, and/or issues related to differences in assay sensitivity in the mouse and human TTR ELISA. While this meganuclease was generally less effective throughout our studies, the conservation of its target site through mice, NHPs, and humans enabled us to evaluate the translatability of this therapeutic strategy across three species.

To evaluate the M2TTR meganuclease in mice—whose target site is absent in the mouse genome—we relied on a strategy used previously to evaluate gene editing in the human *PCSK9* gene in mice.^19,21^ Following preadministration of AAV8.TBG.hTTR(V30M) to provide the NHP and human-conserved meganuclease target site, all three doses of AAV8.TBG.M2TTR led to >94% reductions in hTTR levels derived from the AAV vector. A clearer dose–response was seen at the DNA and RNA levels, with up to 65% editing in the h*TTR* sequence supplied by the vector and >90% editing in the vector-derived RNA sequence.

In the subsequent study, we systemically administered this vector to rhesus macaques. The high level, short-term expression of the meganuclease from this approach resulted in an on-target editing efficiency of up to 45% indels in the *TTR* genomic DNA locus and >80% indels in *TTR* RNA following a dose of 3 × 10^13^ GC/kg. This appears to be consistent with reported hepatocyte transduction efficiencies in NHPs of 70–80% on day 17 following administration of 3 × 10^13^ GC/kg of an AAV8 vector expressing a meganuclease targeting *PCSK9*.^19^ There was persistence of editing between the two liver biopsy time points, and there was a concomitant decrease in serum TTR levels of >95% in both animals administered this vector dose.

We did not see a consistent response in NHPs at the lower dose of AAV8.TBG.M2TTR vector (6 × 10^12^ GC/kg). One macaque (NHP ID 1404128) had an average TTR plateau of 9% (similar to macaques administered the fivefold higher dose) compared to 61% of baseline for the other macaque (NHP ID 1305048). NHP 1305048 showed reduced efficacy across all parameters following the same dose of 6 × 10^12^ GC/kg, including a 1.7- and 4.1-fold reduction in DNA and RNA editing at day 18, respectively (Fig. 3A, B), and a 2.6-fold reduction in nuclease RNA levels (Table 2). A potential explanation for this difference is differential vector gene transfer in the liver either based on the number of hepatocytes transduced or by the level of gene transfer per cell (as suggested by lower RNA levels in this animal). However, the exact reason(s) for this difference is unclear.

Reduced gene transfer following systemic administration typically occurs due to NAbs interacting with the vector capsid.^29^ All macaques were screened as seronegative for anti-AAV8 NAbs (NAb titer <1/5) within 1 month before vector administration.^24,25^ On the day of injection, 3/4 macaques had a NAb titer of 1/5 (Supplementary Fig. S3). Natural fluctuations in AAV8 NAb titers in noninjected animals have been previously described.^30^ While both low-dose animals had a NAb titer of 1/5 on day 0, there was a difference in the magnitude of their response by day 28 (Supplementary Fig. S3). At 4 weeks post-vector administration, the macaque with the reduced editing efficacy (1305048) had a NAb titer of 1/1280, compared to a titer of 1/80 in the other low-dose animal. This larger NAb response at day 28 suggests a rebounding B cell memory response to the AAV8 capsid, which may have reduced vector transduction and efficacy in this macaque.

For the two NHPs administered 3 × 10^13^ GC/kg of AAV8.TBG.M2TTR, we observed an average of 42% indels in genomic DNA, which correlated with 80% editing in *TTR* RNA, and a reduction in serum TTR levels to 4% of baseline. After characterizing the M2TTR indels, we found that this meganuclease generated similar levels of 2 and 3 bp deletions in genomic DNA (Supplementary Fig. S5), with a 3 bp deletion predominating in *TTR* RNA (Supplementary Fig. S6).

In addition to evaluating on-target efficacy, we performed an extensive and unbiased genome-wide detection of off-targets. We previously used this strategy to evaluate a meganuclease targeted to the *PCSK9* gene locus from an AAV vector.^19–21,27^ Compared to the previously evaluated M2PCSK9 meganuclease used for editing within the *PCSK9* gene, the M2TTR meganuclease had off-target events equivalent to some of the self-targeting and short-promoter vectors developed to increase specificity and reduce off-targets without losing efficacy.^21^ Therefore, methods to reduce off-targets by reducing meganuclease expression may be unnecessary for the M2TTR meganuclease.

We observed ∼3% indels in an identified off-target site for the M2TTR meganuclease. This site corresponds to an intergenic region in the rhesus macaque genome, meaning the chances of inadvertently affecting the expression of a gene are low. Furthermore, the risk associated with editing this region is likely limited to rhesus macaques, as this off-target sequence is not found in the human genome. While evaluation of off-target activity in NHPs is helpful to predict potential off-target activity in humans, studies in human cells and/or humanized mice would be required.^20^ The dose of AAV8.TBG.M2TTR used in a human clinical trial could also be reduced from those evaluated here as we observed >95% reductions in TTR levels at 3 × 10^13^ GC/kg. Indeed, current treatment approaches achieve clinically meaningful outcomes when TTR is reduced by 80%.^12,13^ Reducing the vector dose to decrease both on- and off-target efficacy would enhance the safety profile of this approach while retaining its therapeutic efficacy.

While TTR knockdown approaches by either antisense oligonucleotide therapy or RNA interference have recently gained approval for the treatment of ATTR, both approaches have limitations. Inotersen requires weekly administration and routine monitoring for adverse events.^13,15^ Patisiran is administered every 3 weeks IV following a significant premedication protocol to prevent infusion reactions.^12^ Attempts are underway to increase the duration of action of the RNA interference approach with vutrisiran, which is currently in phase 3 clinical trials and is administered quarterly using subcutaneous injection.^31^

In comparison, a gene-editing approach could provide an effective one-time treatment. Another gene-editing approach utilizing liver-targeted LNPs for delivery of a CRISPR-Cas9-based strategy has shown similar reductions of >94% in TTR levels in mice and NHPs.^16,17^ Interim clinical trial data from this approach were recently published in which reductions of 87% (range of 80–96%, *n* = 3 subjects) were achieved in hereditary ATTR patients at day 28 following a dose of 0.3 mg/kg.^18^ As with other LNP-based treatments, this approach also required preadministration of glucocorticoids and anti-histamines; one patient in the trial had an infusion reaction. By delivering the M2TTR nuclease through an AAV vector, our approach capitalizes upon the established safety profile of gene therapy vectors and, similar to our previously published results, the expression of the nuclease is not sustained.^19–21^ Although using this approach for patients with ATTR would require screening for the presence of preexisting NAbs to the vector capsid, this approach results in stable genome editing up to 3 years post-vector administration in NHPs with a similar meganuclease.^20^ We conclude that the significant reduction in serum TTR levels following genomic editing of the *TTR* gene indicates that AAV delivery of a TTR-specific meganuclease could represent an effective treatment for ATTR.

## Supplementary Material

Supplemental data

Supplemental data

Supplemental data

Supplemental data

Supplemental data

Supplemental data

Supplemental data

Supplemental data

Supplemental data
