# Supplementary material for: Treating Transthyretin Amyloidosis via Adeno-Associated Virus Vector Delivery of Meganucleases
Source: Hum Gene Ther. 2022 Nov 14;33(21-22):1174–86. doi: 10.1089/hum.2022.061 (PMC9700363; doi:10.1089/hum.2022.061)
Supplement: Supplemental data [file Supp_FigS1.pdf]

SUPPLEMENTAL DATA

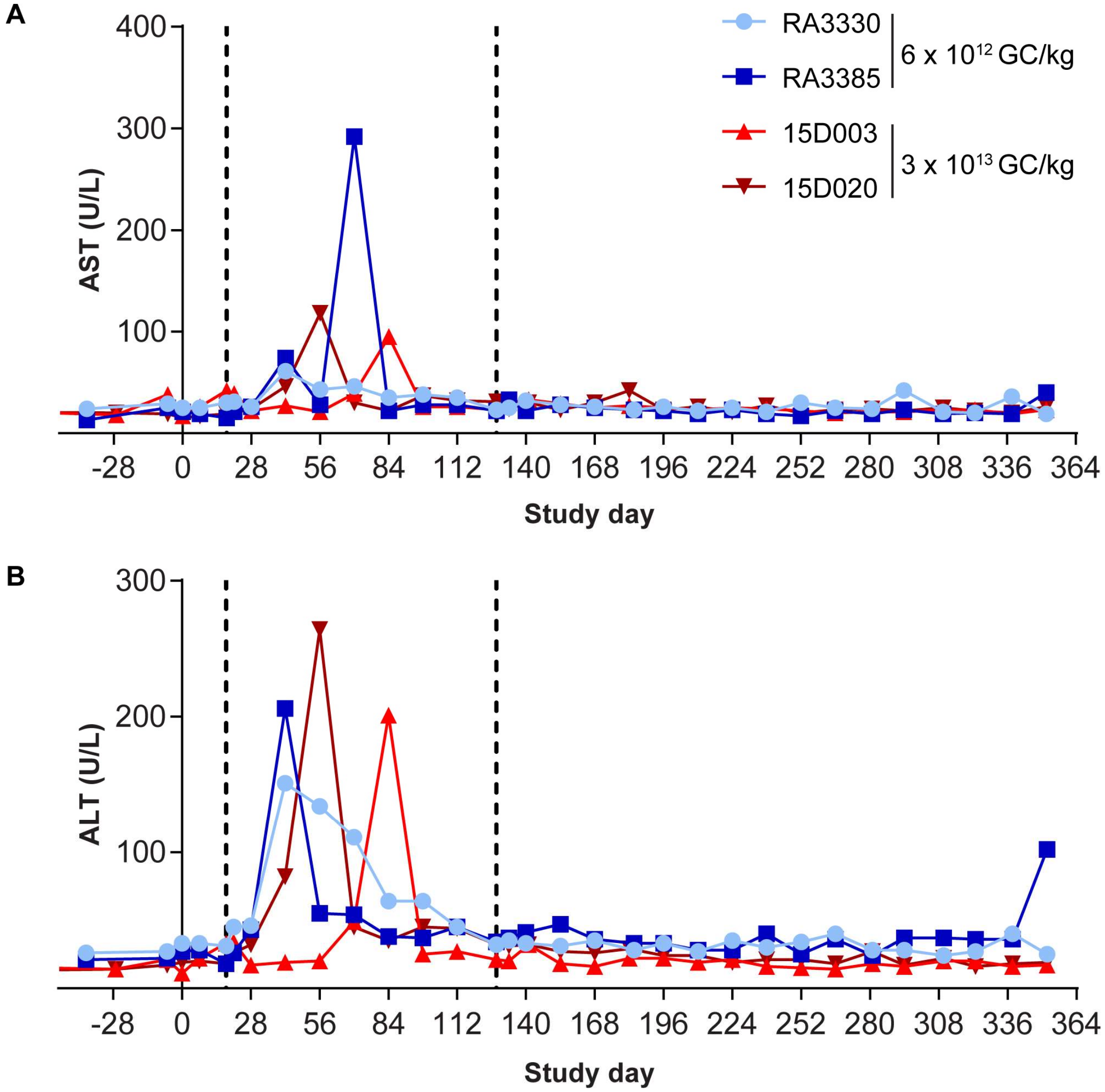

Supplemental Figure S1. Liver function tests following systemic administration of AAV8.TBG.M1TTR.

Rhesus macaques were IV administered $6x10^{12}$ or $3x10^{13}$ genome copies (GC)/kg AAV8.TBG.M1TTR. We performed liver biopsies on day 18 and 128 post-vector administration (indicated by the dotted lines). Animals were necropsied one-year post-vector administration. (A) Aspartate transaminase (AST) and (B) alanine transaminase (ALT) levels were evaluated throughout the in-life phase of the study.
