# Supplementary material for: Treating Transthyretin Amyloidosis via Adeno-Associated Virus Vector Delivery of Meganucleases
Source: Hum Gene Ther. 2022 Nov 14;33(21-22):1174–86. doi: 10.1089/hum.2022.061 (PMC9700363; doi:10.1089/hum.2022.061)
Supplement: Supplemental data [file Supp_FigS3.pdf]

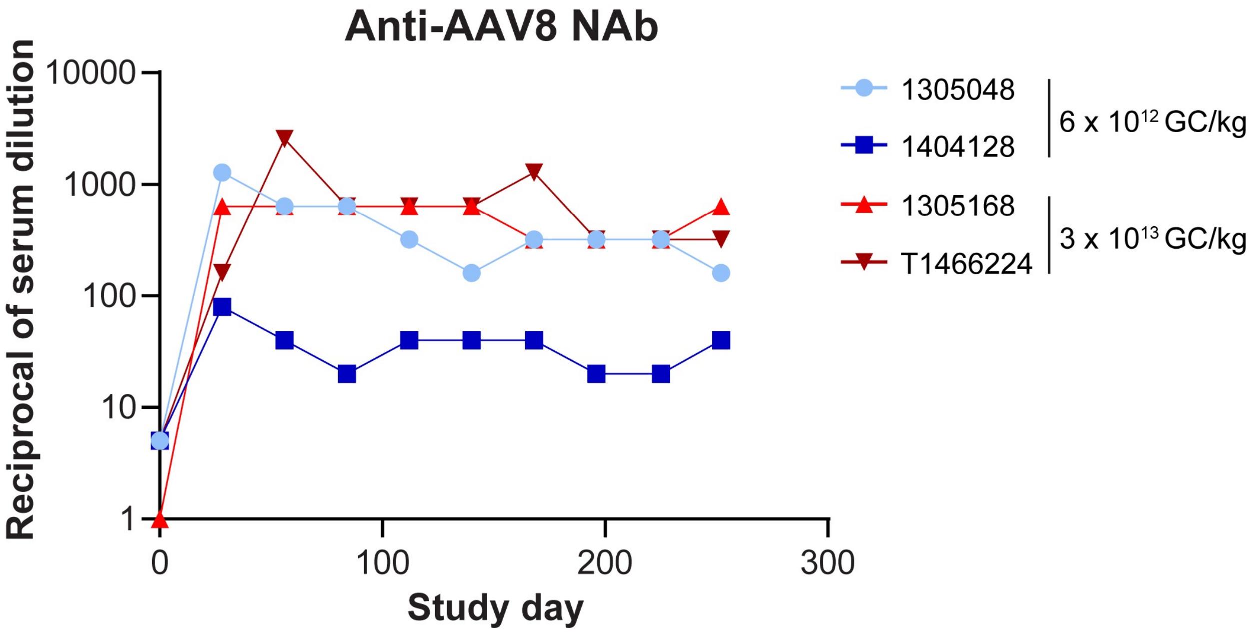

Supplemental Figure S3. Anti-AAV8 NAb levels in NHPs following systemic administration of AAV8.TBG.M2TTR.

Rhesus macaques were IV administered $6x10^{12}$ or $3x10^{13}$ genome copies (GC)/kg AAV8.TBG.M2TTR. We collected blood at selected time points to measure anti-AAV8 NAb in serum. Values are presented as the reciprocal of the serum dilution (limit of detection of the assay is 5). AAV: adeno-associated virus; NAb: neutralizing antibody.
