# Supplementary material for: Treating Transthyretin Amyloidosis via Adeno-Associated Virus Vector Delivery of Meganucleases
Source: Hum Gene Ther. 2022 Nov 14;33(21-22):1174–86. doi: 10.1089/hum.2022.061 (PMC9700363; doi:10.1089/hum.2022.061)
Supplement: Supplemental data [file Supp_FigS4.pdf]

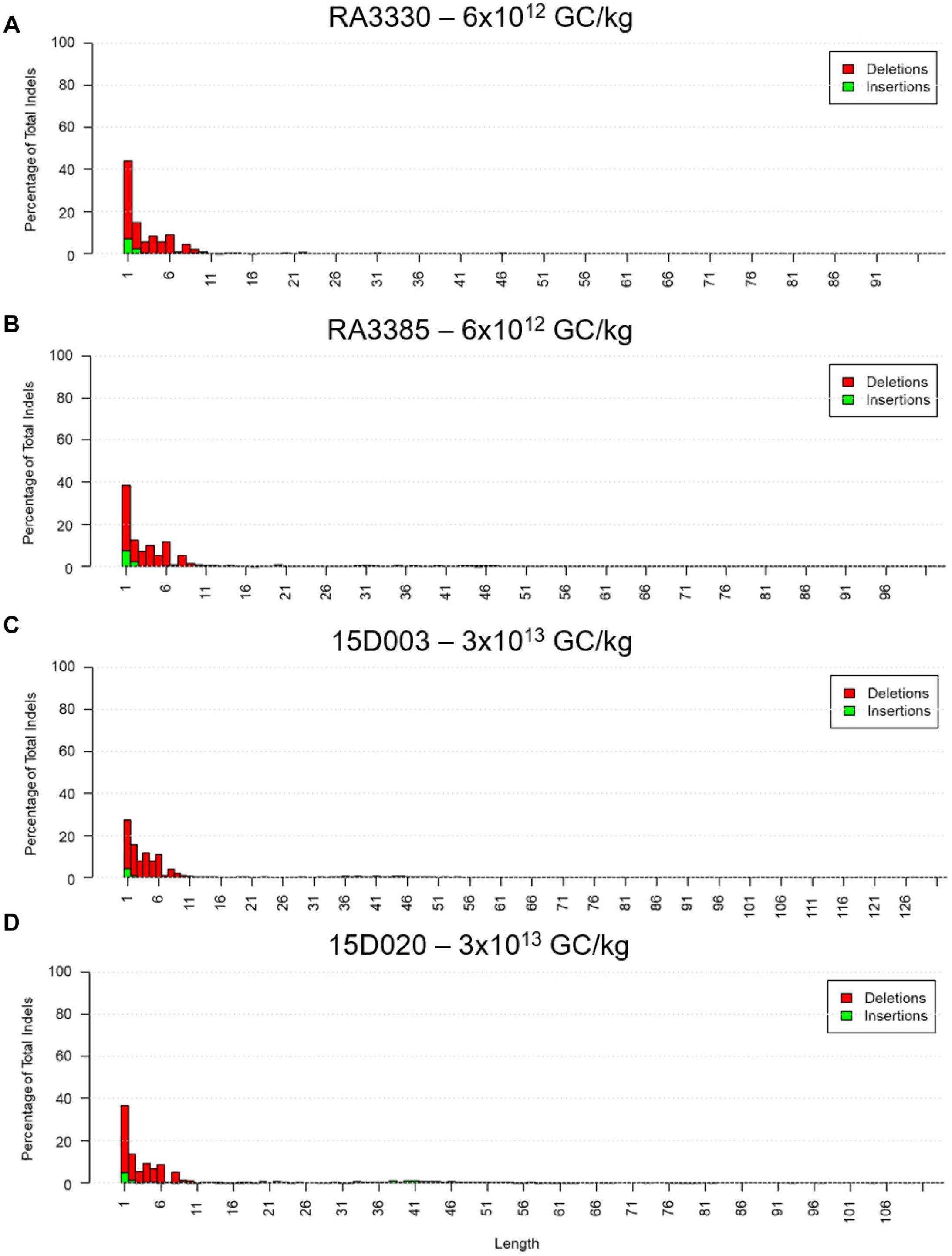

Supplemental Figure S4. Indel pattern characterization in genomic DNA following systemic administration of AAV8.TBG.M1TTR.

Rhesus macaques were IV administered $6x10^{12}$ or $3x10^{13}$ genome copies (GC)/kgAAV8.TBG.M1TTR. Liver biopsies were performed on day 18 post-vector administration. Animals administered AAV8.TBG.M1TTR were necropsied one-year post-vector administration. We extracted DNA, performed NGS analysis, and evaluated the distribution of indel length.
