# Supplementary material for: Treating Transthyretin Amyloidosis via Adeno-Associated Virus Vector Delivery of Meganucleases
Source: Hum Gene Ther. 2022 Nov 14;33(21-22):1174–86. doi: 10.1089/hum.2022.061 (PMC9700363; doi:10.1089/hum.2022.061)
Supplement: Supplemental data [file Supp_FigS6.pdf]

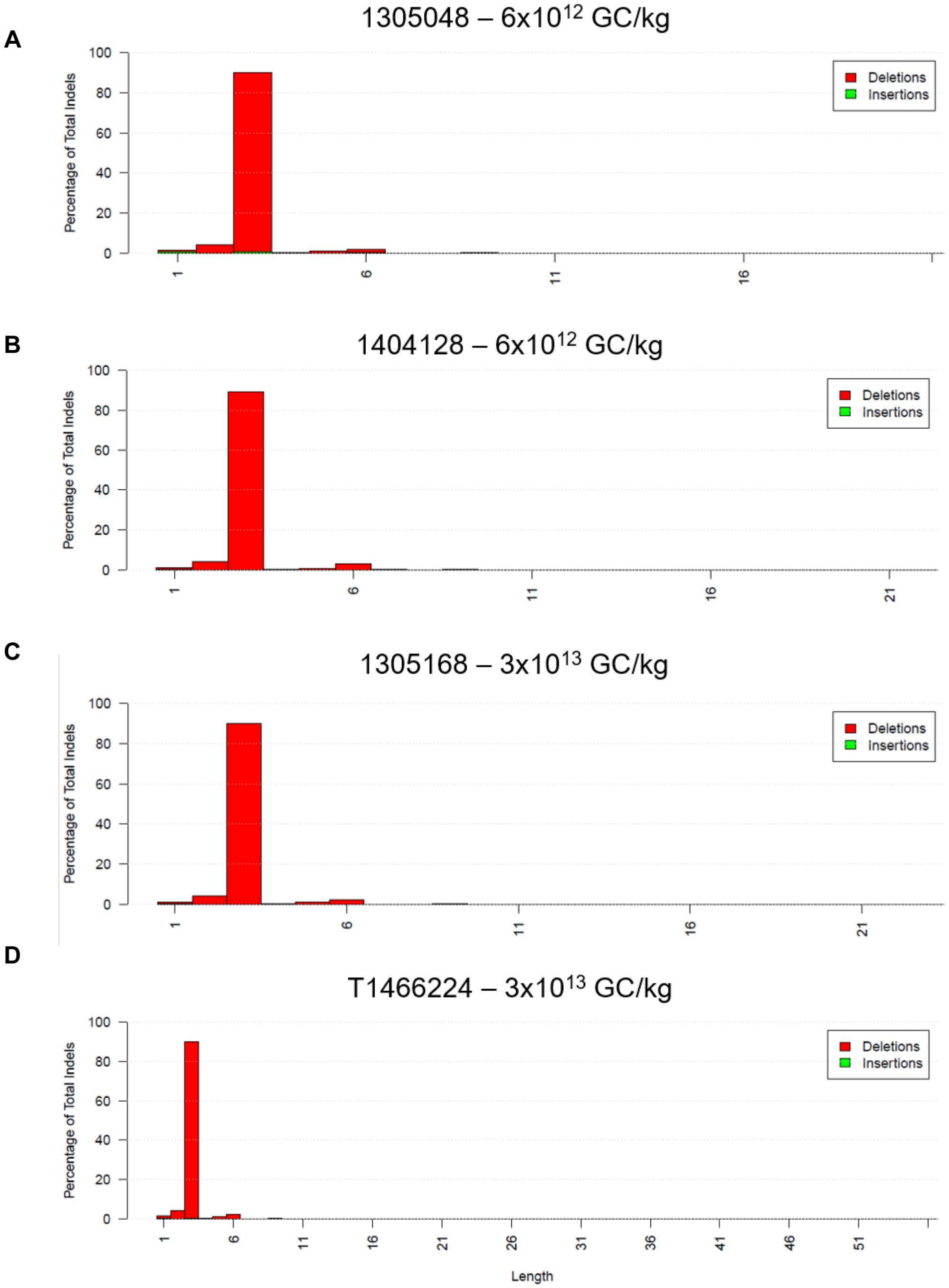

Supplemental Figure S6. Indel pattern characterization in RNA following systemic administration of AAV8.TBG.M2TTR.

Rhesus macaques were IV administered 6×10^{12} or 3×10^{13} genome copies (GC)/kgAAV8.TBG.M2TTR. Liver biopsies were performed on day 18 post-vector administration. We extracted RNA, reverse transcribed it to cDNA, performed NGS analysis, and evaluated the distribution of indel length.
