# Supplementary material for: Treating Transthyretin Amyloidosis via Adeno-Associated Virus Vector Delivery of Meganucleases
Source: Hum Gene Ther. 2022 Nov 14;33(21-22):1174–86. doi: 10.1089/hum.2022.061 (PMC9700363; doi:10.1089/hum.2022.061)
Supplement: Supplemental data [file Supp_FigS7.pdf]

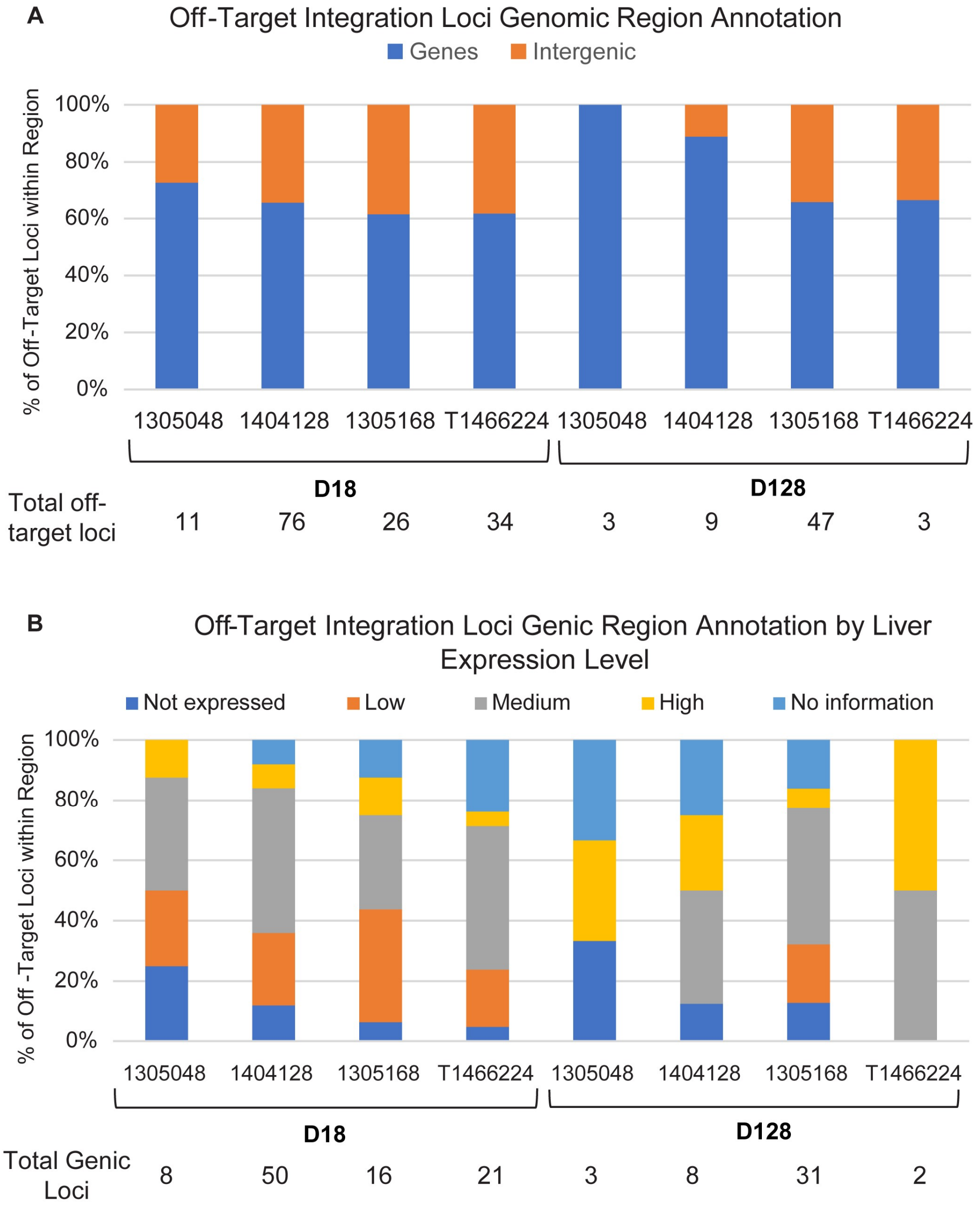

Supplemental Figure S7. Translation of TTR gene editing from mice to NHPs. Rhesus macaques were IV administered $6x10^{12}$ and $3x10^{13}$ GC/kg of AAV8.TBG.M2TTR. We performed liver biopsies on day 18 (D18) and 128 (D128) post-vector administration. We extracted DNA and performed ITR-Seq on each biopsy sample. The genomic location of each unique off-target site was computationally determined from the ITRseq raw sequencing data using our bioinformatics pipeline and then annotated according to the rhesus RefSeqGene annotation. (A) Off-target sites were annotated as being within a gene-coding region (genic) or outside of a gene-coding region (intergenic). (B) Off-target sites within genic regions were further annotated by their respective expression level in the liver. Expression levels were determined by Human Liver expression Normalized Expression (nx) values given for each annotated gene (Human Genome Atlas [www.proteinatlas.org]). Categories were determined as follows: Genes not expressed in liver: 1<nx ; Genes with Low Expression in the liver: 1>=nx<10; Genes with Medium Expression in the Liver: 10<nx>100; Genes with High Expression in the Liver: 100>=nx . NA represents a genomic location in which the species genome is not annotated.
