# Supplementary material for: Treating Transthyretin Amyloidosis via Adeno-Associated Virus Vector Delivery of Meganucleases
Source: Hum Gene Ther. 2022 Nov 14;33(21-22):1174–86. doi: 10.1089/hum.2022.061 (PMC9700363; doi:10.1089/hum.2022.061)
Supplement: Supplemental data [file Supp_FigS8.pdf]

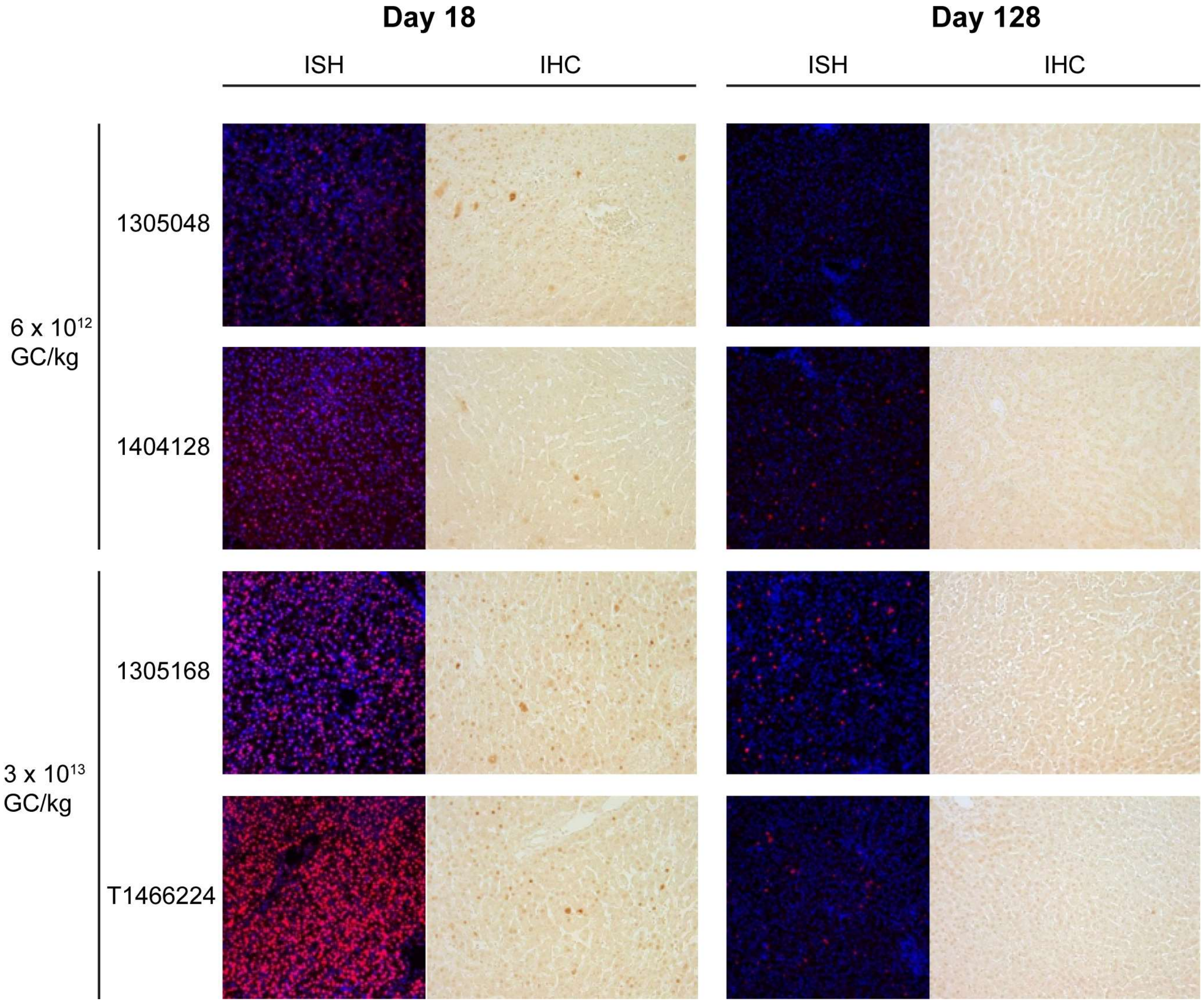

Supplemental Figure S8. ISH for nuclease RNA in liver following systemic administration of AAV8.TBG.M2TTR.

Rhesus macaques were administered IV with $6x10^{12}$ and $3x10^{13}$ genome copies (GC)/kg of AAV8.TBG.M2TTR. We performed liver biopsies on day 18 and day 128 post-vector administration. *In situ hybridization* (ISH) and immunohistochemistry (IHC) staining were performed on liver sections to detect the presence of nuclease. In ISH sections, nuclease RNA is shown with red staining and sections were counterstained with the nuclear stain DAPI (blue).
