# Supplementary material for: Treating Transthyretin Amyloidosis via Adeno-Associated Virus Vector Delivery of Meganucleases
Source: Hum Gene Ther. 2022 Nov 14;33(21-22):1174–86. doi: 10.1089/hum.2022.061 (PMC9700363; doi:10.1089/hum.2022.061)
Supplement: Supplemental data [file Supp_FigS9.pdf]

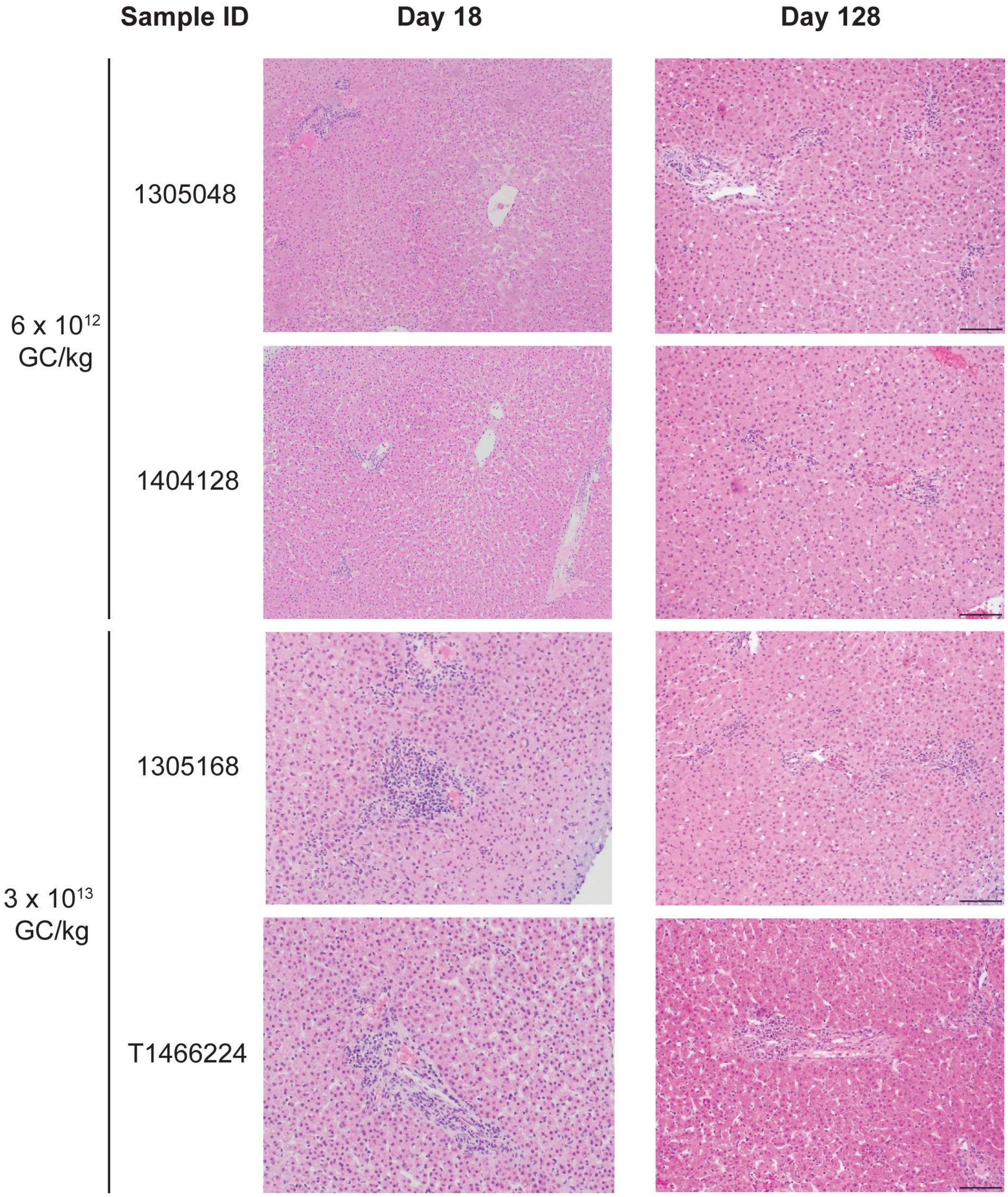

Supplemental Figure S9. Minimal-to-mild histopathology findings in liver following systemic administration of AAV8.TBG.M2TTR.

Rhesus macaques were administered IV with 6x10^{12} and 3x10^{13} genome copies (GC)/kg of AAV8.TBG.M2TTR. We performed liver biopsies on day 18 and day 128 post-vector administration. H&E staining was performed, and liver sections were evaluated by a board-certified veterinary pathologist. Images were taken to exemplify the finding.
